# Low-temperature poly-Si nanowire junctionless devices with gate-all-around TiN/Al_2_O_3_ stack structure using an implant-free technique

**DOI:** 10.1186/1556-276X-7-339

**Published:** 2012-06-22

**Authors:** Chun-Jung Su, Tzu-I Tsai, Horng-Chih Lin, Tiao-Yuan Huang, Tien-Sheng Chao

**Affiliations:** 1 Nano Facility Center, National Chiao Tung University, Hsinchu, 300, Taiwan; 2 Department of Electronics Engineering and Institute of Electronics, National Chiao Tung University, Hsinchu, 300, Taiwan; 3 Department of Electrophysics, National Chiao Tung University, Hsinchu, 300, Taiwan; 4 National Nano Device Laboratories, Hsinchu, 300, Taiwan

**Keywords:** Accumulation mode, Gate-all-around, Junctionless, Low-temperature poly-Si, Nanowire

## Abstract

In this work, we present a gate-all-around (GAA) low-temperature poly-Si nanowire (NW) junctionless device with TiN/Al_2_O_3_ gate stack using an implant-free approach. Since the source/drain and channel regions are sharing one *in situ* phosphorous-doped poly-Si material, the process flow and cost could be efficiently reduced. Owing to the GAA configuration and small volume of NW channels, the fabricated devices with heavily doped channels display superior switching behaviors and excellent immunity to short-channel effects. Besides, the negative fixed charges in Al_2_O_3_ are found to be helpful to obtain desirable positive threshold voltages for the n^+^-poly-Si channel devices. Thus, the simple and low-cost fabrication method along with excellent device characteristics makes the proposed GAA NW transistor a promising candidate for future 3-D electronics and system-on-panel applications.

## Background

With the aggressive downscaling of transistor dimensions to increase the speed and density of transistors on an integrated circuit, nanowire (NW) field-effect transistors (FETs) are considered as one of the most promising device architectures to meet the requirements [1-3]. Hence, a plethora of researches focusing on NW-based devices especially with multiple-gated structure has been widely explored [3-6]. Owing to the inherent tiny volume of NW, it enables better gate controllability for overcoming the short-channel effects over the planar FETs because the electrostatic potential in the ultrathin channel can be effectively controlled so that the channel suffers less electrical interference from the drain [3,5]. Concurrently, the control on junction doping profiles of source/drain (S/D)-to-channel regions becomes extremely challenging in nanoscale regimes. In line with this, junctionless (JL) devices have been proposed to cope with the doping profile issue [7,8]. In a JL structure, the dopant type and concentration are the same all the way from the source, channel to drain. Such JL transistor is operated as an accumulation-mode device and basically a gated resistor in the on-state, while it can be switched off by full depletion of carriers in the channel by the gate. Since no conventional p-n junctions are formed in it, the JL scheme can relieve the stringent formation technique of the ultra-shallow or ultra-abrupt junction, thus simplifying the fabrication process. Furthermore, the conduction mechanism of such scheme is via currents passing through the body of the channel, and accordingly, the most important criteria of this scheme is that the channel layer must be thin enough so it can be entirely depleted by the potential difference exerted by the gate [7]. In this work, we present a junction-free NW device with gate-all-around (GAA) TiN/Al_2_O_3_ stack using one *in situ* doped poly-Si material for both the channel and S/D regions without any implant process. Due to the GAA structure together with the small body, the gate is capable of depleting the heavily doped channel thoroughly to switch the device off, thus obtaining a high on/off current ratio as well as good on-state performance. In addition, most high-*κ* oxides have positive fixed charges, except that Al_2_O_3_ has negative fixed charges [9]. By taking advantage of this feature, we investigate the utilization of Al_2_O_3_ as the gate dielectric for the n^+^-poly-Si channel device and expect that the proposed JL device could be desirably operated in a positive threshold voltage (*V*_th_) range for favoring its applications in logic circuits and memory devices.

## Methods

### Device fabrication and experiment

**Figure 1:**
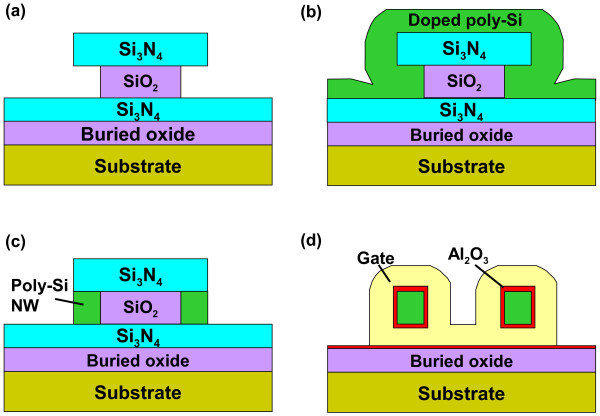
**Schematic process flow of the proposed GAA JL NW device.** (**a**) A sandwich stack of nitride/TEOS oxide/nitride layers was sequentially deposited and then patterned on a Si substrate capped with a 200-nm-thick wet oxide. Next, selective lateral etching of the TEOS oxide layer was performed using diluted HF solution to form a sub-100-nm cavity underneath both sides of the top nitride. (**b**) Deposition of an *in situ* phosphorus-doped poly-Si layer. (**c**) The NW doped channels (denoted as DC) and S/D regions were defined simultaneously. (**d**) After etching off the nitride and oxide surrounding the NW channels, the high-*κ*/metal gate stack structure was formed using the atomic-layer deposition system to conformally deposit 10-nm Al_2_O_3_ and 10-nm TiN films onto the NW channel.

**Figure 2:**
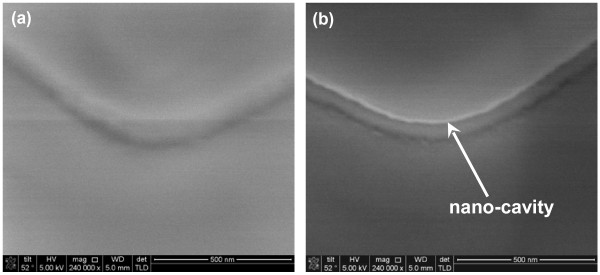
**Tilted SEM images of the dielectric stack.** (**a**) Before lateral etching and (**b**) after lateral etching of 60 s with diluted HF solution showing the formation of nano-cavity.

**Figure 3:**
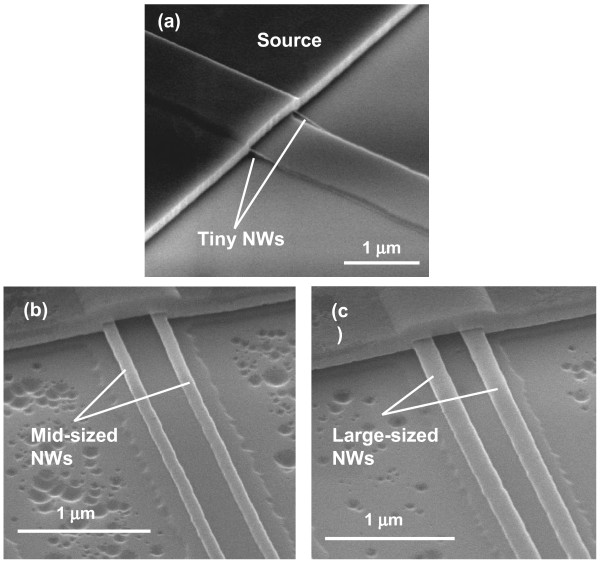
**52° tilted SEM images of different sized NW devices before the gate stack formation.** (**a**) A tiny NW device showing NWs exposed on both sides of the temporary dielectric step, (**b**) a mid-sized NW device, and **(c)** a large-sized NW device.

**Figure 4:**
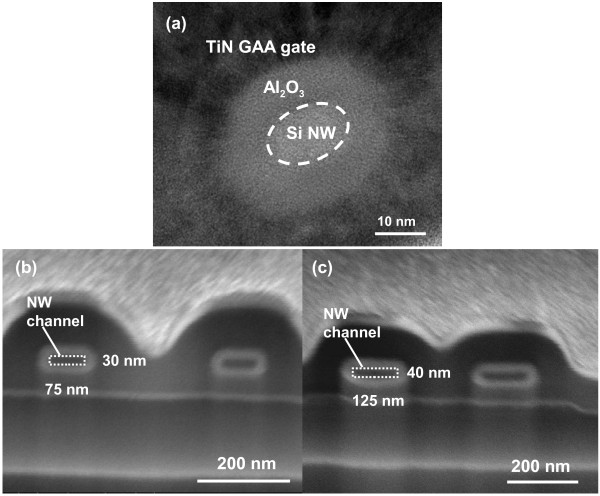
**TEM and SEM images of a DC device.** (**a**) Cross-sectional TEM image of a DC device with tiny NW covered with Al_2_O_3_/TiN GAA stack. 52° tilted SEM images of (**b**) a mid-sized DC device with a NW cross section of 75 × 30 nm and (**c**) a large-sized DC device with a NW cross section of 125 × 40 nm.

The key fabrication process flow of the proposed GAA JL NW FET is briefly described in Figure 1. First, a sandwich stack of nitride/tetraethyl orthosilicate (TEOS) oxide/nitride layers was sequentially deposited and then patterned on a Si substrate capped with a 200-nm-thick wet oxide. Next, the formation of a sub-100-nm cavity underneath both sides of the top nitride was fulfilled by selective lateral etching of the TEOS oxide layer using diluted hydrofluoric (HF) solution (HF:H_2_O = 1:100) as shown in (Figure 1a). The scanning electron microscopic (SEM) images before and after cavity formation are depicted in Figure 2. With 60-s etching time, a vivid shape of cavity was formed and served as the template for the formation of NW channels. An *in situ* phosphorus-doped poly-Si layer was then deposited at 550°C using a mixture of SiH_4_/PH_3_ gases in the LPCVD system (Figure 1b), followed by an anisotropic plasma etching to define the NW doped channels (denoted as DC) and S/D regions simultaneously to form the structure without junctions (i.e., n^+^-n^+^-n^+^), as illustrated in (Figure 1c). Note that the conventional control devices (i.e., n^+^-i-n^+^) were also processed along with a similar flow but using undoped poly-Si as the channel (denoted as undoped channel, UC), while the S/D regions were formed with the same *in situ* doped poly-Si as in the DC device. To implement the GAA structure, the sandwich stack layers were removed to suspend the NW channels, as revealed in Figure 3 with various volumes of NWs. Subsequently, the high-*κ*/metal gate stack structure was formed using the atomic-layer deposition system to conformally deposit 10-nm Al_2_O_3_ and 10-nm TiN films onto the NW channel, as shown in (Figure 1d). Note that the final thickness of the TiN gate electrode is 150 nm by adding another 140-nm TiN film through sputtering. After patterning the gate electrode, depositing passivation layer, and standard metallization procedure, the fabrication of NW devices was accomplished. (Figure 4a) shows the cross-sectional transmission electron microscopic (TEM) image of a GAA NW device with NW channel dimensions of about 11 × 15 nm. In this study, NW channels with the cross section of such dimensions for DC and UC devices are used for characterization unless mentioned otherwise.

In this study, Hall measurements were conducted on a blanket *in situ* doped poly-Si thin film of 3,000 Å; the carrier concentration of PH_3_ with 15 sccm flow rate was found to be around 1 × 10^20^ cm^−3^. However, the practical active carrier concentration in the NW channels of the fabricated devices would be much lower than the result of Hall measurements due to the effects of film volume [10] and donor deactivation occurring in the Si NW structure [11].

## Results and discussion

**Figure 5:**
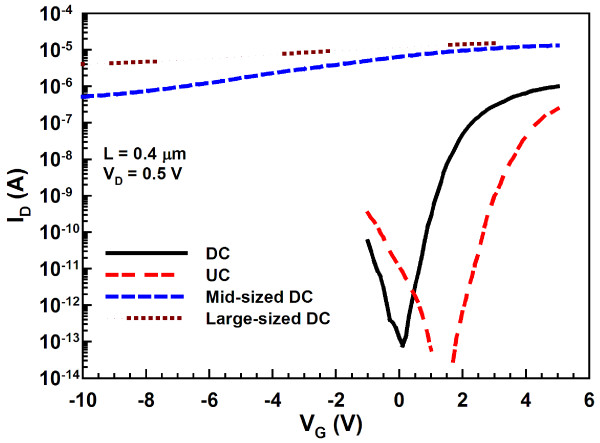
**Transfer characteristics of the DC and UC NW devices.** Mid-sized (75 × 30 nm) and large-sized (125 × 40 nm) DC NW devices are also plotted for comparison. All the devices characterized here are with channel length of 0.4 μm.

**Figure 6:**
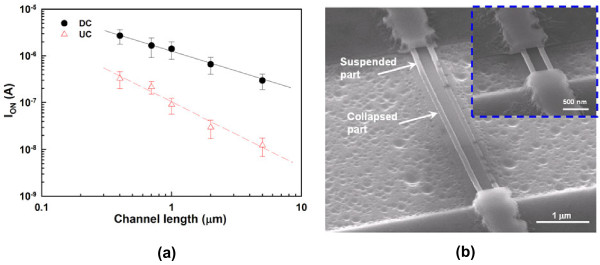
**On currents and 52° tilted SEM image of a NW device.** (**a**) On currents, extracted at *V*_G_ − *V*_th_ = 2 V and *V*_D_ = 0.5 V, as a function of channel length for DC and UC devices. (**b**) 52° tilted SEM image of a NW device with *L* = 5 μm showing collapse of NWs in the central channel region. The inset shows a NW device with *L* = 1 μm depicting normal suspension of NWs between the S/D regions.

Transfer characteristics of the DC and UC NW FETs are depicted in Figure 5, in which another two DC NW devices with larger channel cross sections are also compared to illustrate the volume effect on the switching behaviors. The cross-sectional dimensions of the NWs for these two larger devices are 75 × 30 nm (mid-sized DC) and 125 × 40 nm (large-sized DC), respectively, as shown in (Figure 4b,c). Apparently, more aggravated switching properties are observed for larger NW channels among the three splits of DC devices. Owing to the conduction mechanism of the junctionless scheme, the bulk conduction path in the channel is not apt to be fully depleted as the channel cross section increases [7]. Therefore, for the mid-sized and large-sized DC devices, they cannot be effectively turned off by the gate, even though a high gate bias (*V*_G_) of −10 V is applied as revealed in Figure 5. On the other hand, the DC device with small NW channels displays superior switching behaviors with a subthreshold swing of 210 mV/dec and on/off current ratio of 7 × 10^6^, outperforming its UC counterpart with the current ratio of 2 × 10^6^. In the above comparison, the on current (*I*_ON_) is defined as the drain current (*I*_D_) at *V*_G_ − *V*_th_ = 2 V, while the off current (*I*_OFF_) is the minimal *I*_D_. (Figure 6a) compares the on currents, extracted at *V*_G_ − *V*_th_ = 2 V and *V*_D_ = 0.5 V from both types of devices, as a function of channel length (*L*). In our device structure, the channel length is defined as the spacing between the S/D regions. The *I*_ON_ is evidently boosted in the DC devices, especially as the channel length is long. This is attributed to the much larger cross section available for carrier flow, leading to the reduction in the channel resistance for the DC devices [7,8]. Besides, the carriers available for conduction in the DC devices also outnumber those in the UC counterparts. Nevertheless, for shorter channels, the enhancement is not so pronounced. We ascribe this to two plausible reasons. For one, the series resistance in the S/D regions (*R*_SD_) for the DC devices is still high and could contribute to this trend as the channel length decreases, although *R*_SD_ has been demonstrated to be reduced in the junctionless scheme as compared with its counterpart with junctions [8,12]. In order to further boost the *I*_ON_ enhancement, the S/D regions should be silicided or doped with higher concentration. Besides, as can be seen in (Figure 6b), due to the small and long NW structure, the region of NW channel located away from the supporting source/drain ends tends to tumble down and might touch the substrate surface. However, for a shorter-channel device, the NWs normally suspend between the S/D regions as depicted in the inset. Thus, after the gate stack formation, the actual gate structure in the central part of a long channel may not be in a perfect gate-all-around configuration. Since the conduction mechanism in the DC and UC transistors is mainly through bulk and surface paths, respectively, the central region of the channel would be induced with fewer carriers for the UC device. As a result, the *I*_*O*N_ enhancement for the DC transistors becomes larger as channel length increases, and this result may also imply that the DC device is less susceptible to gate configuration in the on-regime.

**Figure 7:**
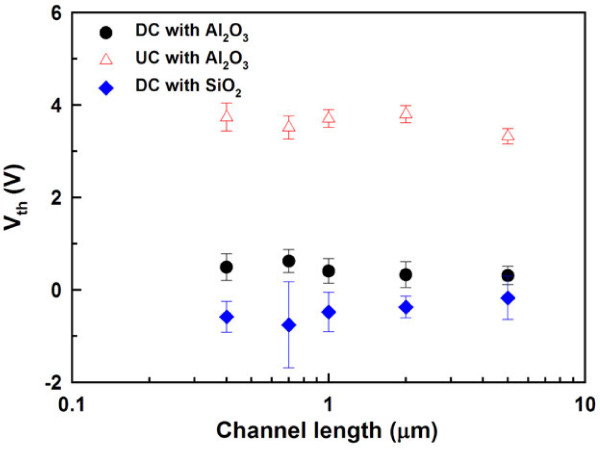
**Threshold voltage as a function of channel length for DC and UC devices.** DC devices with GAA TiN/SiO_2_ gate stack are also plotted for comparison.

Figure 7 shows the threshold voltage as a function of channel length for both types of devices. The smaller *V*_th_ values found in the DC devices are a result of the accumulation-mode operation. Due to the large work-function difference between the n^+^-doped NW channels and TiN gate, reasonable *V*_th_ values can be achieved for the DC devices. Furthermore, as compared with their counterparts with SiO_2_ gate dielectric, positive *V*_th_ values found in the DC devices with Al_2_O_3_ could be ascribed to the effect of negative fixed charges contained in Al_2_O_3_. Besides, no obvious *V*_th_ roll-off behaviors are observed in both types of devices with Al_2_O_3_, suggesting the effectiveness of the GAA structure and high-*κ* gate dielectric exploited.

## Conclusions

In summary, we have reported the fabrication and experimental investigation of the junction-free GAA NW device with TiN/Al_2_O_3_ gate stack structure by employing an implant-free method. From the results of the electrical characterizations, a sufficiently small cross section of the NW channels is essential to obtain superior on/off current ratio for heavily doped channel devices. The fabricated DC devices also show excellent *V*_th_ roll-off properties and boosted on-state performance especially as the channel length increases. The adoption of Al_2_O_3_ as the gate dielectric shifts the *V*_th_ to a positive value and thus is conducive to acquiring desirable *V*_th_. With its low cost and straightforward processing, we believe that the proposed GAA NW JL transistor architecture is promising for future 3-D electronics and system-on-panel applications.

## Competing interests

The authors declare that they have no competing interests.

## Authors’ contributions

CJ carried out the SEM and TEM characterization, performed the electrical analysis, and drafted the manuscript. TI fabricated the samples and carried out the electrical characterization. HC participated in the design and coordination of the study. All authors read and approved the final manuscript.
